# Plastaumatic: Automating plastome assembly and annotation

**DOI:** 10.3389/fpls.2022.1011948

**Published:** 2022-11-03

**Authors:** Wenyi Chen, Sai Reddy Achakkagari, Martina Strömvik

**Affiliations:** Department of Plant Science, McGill University, Sainte-Anne-de-Bellevue, QC, Canada

**Keywords:** plastome, organellar genome, chloroplast genome, sequence assembly, plant

## Abstract

Plastome sequence data is most often extracted from plant whole genome sequencing data and need to be assembled and annotated separately from the nuclear genome sequence. In projects comprising multiple genomes, it is labour intense to individually process the plastomes as it requires many steps and software. This study developed *Plastaumatic* - an automated pipeline for both assembly and annotation of plastomes, with the scope of the researcher being able to load whole genome sequence data with minimal manual input, and therefore a faster runtime. The main structure of the current automated pipeline includes trimming of adaptor and low-quality sequences using *fastp*, *de novo* plastome assembly using *NOVOPlasty*, standardization and quality checking of the assembled genomes through a custom script utilizing *BLAST+* and *SAMtools*, annotation of the assembled genomes using *AnnoPlast*, and finally generating the required files for NCBI GenBank submissions. The pipeline is demonstrated with 12 potato accessions and three soybean accessions.

## Introduction

Plastids are essential organelles in plant cells as they host the vital reactions of photosynthesis (as chloroplasts), store starch and sugars (amyloplasts), lipids and oils (elaioplasts), as well as pigments (chromoplasts). All differentiated plastid types develop from the proplastid. Just like the mitochondrion, the (pro)plastid has its own genome, also known as the plastome. The plastome of most land plants is relatively conserved in size and structure – a circular molecule in the size range of 120,000 to 170,000 base pairs. It usually consists of four structural regions including one large single copy (LSC), one small single copy (SSC), and two inverted repeat regions (IRa and IRb) (Chung et al., 2006). Being highly conserved across species, the genetic information contained in the plastome could hold keys to a better understanding of plant adaptation, as well as crop improvement and breeding. Being generally inherited maternally (just like the mitogenome), the plastome is often extensively studied in phylogenetic analyses of plants (McCauley et al., 2007).

Plastome assembly typically includes the following manually initiated steps: the trimming of adaptors and low-quality sequences from whole genome sequencing data using tools such as *Trimmomatic* (Bolger et al., 2014), *de novo* plastome assembly using the most popular tool *NOVOPlasty* (Dierckxsens et al., 2017), or GetOrganelle (Jin et al., 2020) and annotation of the assembled genomes with well-annotated reference plastomes using *PGA* (Qu et al., 2019) or *GeSeq* (Tillich et al., 2017), where the running of each tool mentioned above requires a written script specifying paths of input and output files, the executing commands and modified parameters.

As more and more projects sequence multiple plant genomes for comparison and need to assemble the corresponding plastomes (Achakkagari et al., 2020; Achakkagari et al., 2021; Camargo Tavares et al., 2022; Hoopes et al., 2022), the time spent on tedious and repeated manual input and sorting can be avoided if the process was automated. An automated workflow for fast and accurate assembly as well as annotation of plastome sequences from raw whole (nuclear) genome sequencing data is needed.

Currently there are no automated pipelines for the assembly and annotation of the plastomes. For example, the pipeline *NOVOWrap*, (Wu et al., 2021a) is available publicly and can assemble and standardize the plastome sequences, however it does not incorporate trimming and annotation methods in the pipeline. The Fast-Plast (McKain and Wilson, 2017) is another similar tool, which however also does not incorporate annotation in its pipeline.

In the current study an automated pipeline for both assembly and annotation of plastomes was developed, with the scope of the researcher being able to load whole (nuclear) genome sequence data from any number of genotypes, species, or related organisms at a time, with minimal manual input, and therefore a faster completion rate. The pipeline is demonstrated with two sets of plant sequence data: three soybean accessions, and 12 potato accessions, and shows substantially faster completion than manual assembly.

## Methods

**Figure 1:**
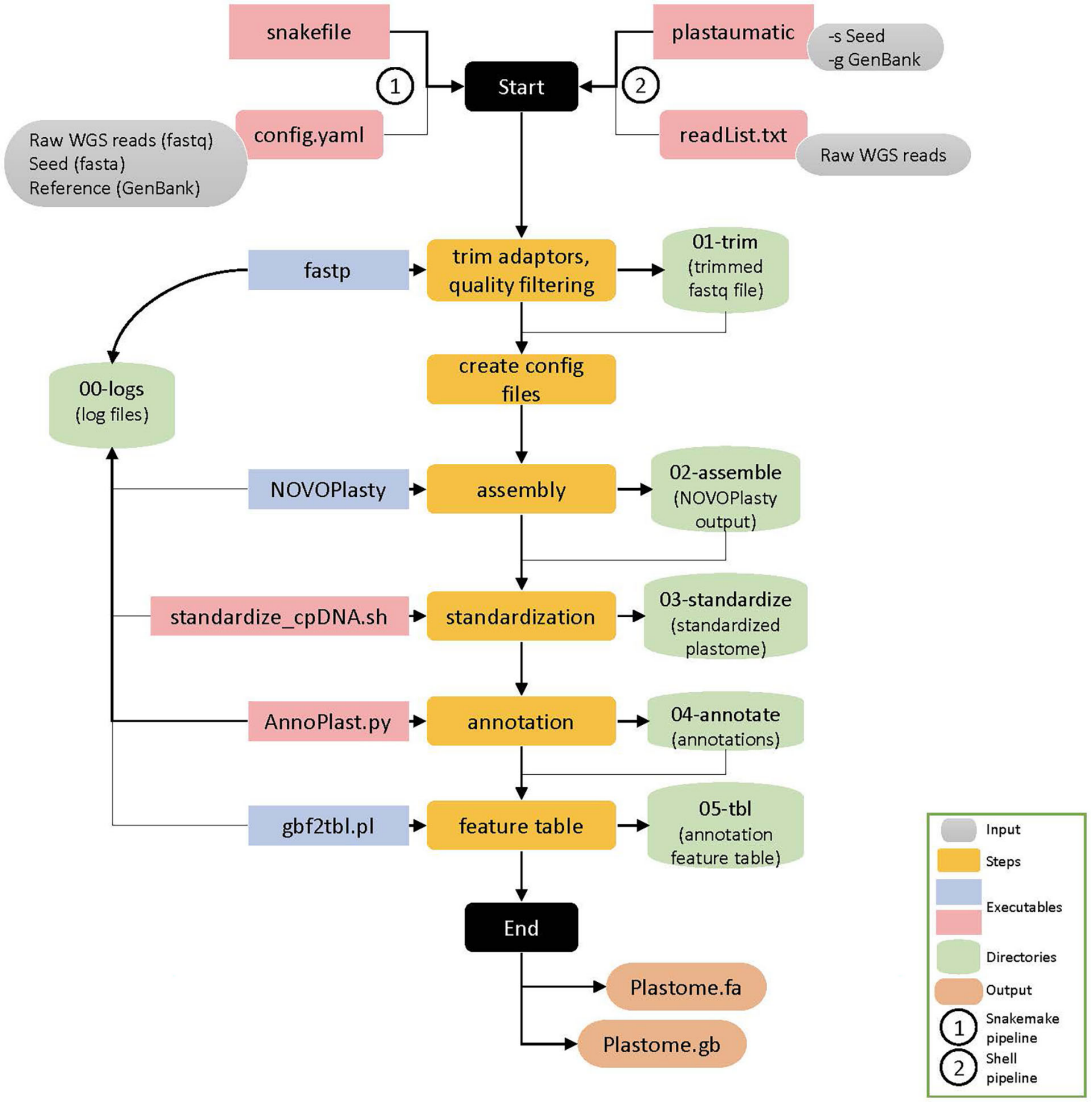
Schematic representation of the *Plastaumatic* plastome assembly pipeline with details. There are six steps involved in the *Plastaumatic* pipeline i.e., trimming, creating config file for assembly, *de novo* assembly, standardization, annotation, and generating a feature table file. The pipeline can be run using Snakemake or a shell script, both of which produce the same output. The input data required for the *Plastaumatic* is the raw WGS reads in *fastq* format, a seed file in *fasta* format, and an annotation file in *GenBank* format. The pipeline creates multiple directories as detailed in the figure to store all the output.

The automation of the pipeline was achieved through Snakemake, a specification language built on Python (Mölder et al., 2021). A snakefile outlining rules that describe steps in a workflow defining how to obtain output files from input files. Dependencies between rules are determined automatically according to the manner the snakefile was written. Upon executing the snakefile, Snakemake can then run through all described steps in the workflow at once by taking the output files from an upstream rule and automatically feed them into the next rule. This automated pipeline for plastome assembly and annotation was made automatic through specifying the connections of input and output files for each program to those of the next and previous program. The automated processes in this pipeline specified in the main executable snakefile include six steps: quality trimming by *fastp*, generating input config files for *de novo* assembly, *de novo* assembly by *NOVOPlasty*, standardization of the assembled genomes using a custom script, annotation of the assembled plastomes by *AnnoPlast.py*, and GenBank to feature table conversion using *gbf2tbl.pl* script from NCBI tools (**Figure 1**).

### User input

In order to execute the pipeline, the computing systems used need to have Snakemake installed (https://snakemake.readthedocs.io/en/stable/getting_started/installation.html). Executing the pipeline requires a configuration file from the user. The configuration file requests the paths of the forward and reverse raw reads in *fastq* format (compressed or uncompressed), a seed sequence in *fasta* format from a closely related reference plastome (usually a well conserved gene), an annotated reference plastome in GenBank format, paths to the main executable script *NOVOPlasty*, path to the *Plastaumatic* repository, and a plastome assembly size range.

### Execution

The pipeline can be executed by simply executing the *snakefile* from any desired directory. An additional wrapper script written in shell is also available for running the *Plastaumatic* pipeline and it does not require to have Snakemake installed. This can be executed by running *plastaumatic -s <seed.fa> -g <reference.gb> -r <range> -f <fof.txt> -n <NOVOPlasty4.3.1.pl>*. Here, paths to the raw sequencing data are provided in a simple text file (*fof.txt*). In both methods, upon execution a user-specified *prefix* directory is created for each sample and all the rules are run. Upon a successful run, links to plastome assembly and annotation of each sample are created under their *prefix* directory.

### Pipeline

The first rule in *snakefile* is to perform adaptor removal and quality filtering using an ultra-fast FASTQ pre-processor *fastp* (Chen et al., 2018). We chose *fastp* because it provides faster performance and additional functionality such as automatic detection of adapter sequences and subsampling a fraction of reads from the input. We have tested different subsets for the filtered data (1, 5, 10, 15 million reads) and chose the optimal value of 10 million reads for this pipeline. All the other parameters are set to default for *fastp*. In the next rule, a config file required by *NOVOPlasty* is created by adding filtered read paths, path to seed file, range, and other parameters. A *de novo* plastome assembly is then performed using *NOVOPlasty* in the following rule. A successful *NOVOPlasty* run will create either a single circular assembly or two circular assemblies (Option 1 and 2) with different orientation of the SSC region. Also, the assemblies are not standardized, meaning the *fasta* file can start from any region in the plastome. Standardization of the assembly is necessary for accurate downstream analyses such as annotation and multiple plastome comparisons. Since no suitable publicly available tool was found for this process, a custom written shell script *standardize_cpDNA.sh* which uses *BLAST+* (Camacho et al., 2009) and *SAMtools* (Danecek et al., 2021) is used for standardization. The plastome assembly is first aligned with itself to get the repeat sequences and to locate the four main regions of a plastome (LSC, SSC, IRa, and IRb). The assembly is split at these four regions and joined together to make a standardized assembly in the form of LSC-IRb-SSC-IRa. If *NOVOPlasty* produces two assembly options, one option is selected based on the SSC orientation of the reference plastome and is used for subsequent analyses. In some rare cases, *NOVOPlasty* outputs ambiguous bases (non-ACTG) in the assembly. These are also corrected from the assembly using reads to get a final clean assembly. In the next rule, the standardized assembly is used for the annotation by *AnnoPlast.py*. Current plastome annotation tools either does not annotate some features or improperly annotate feature boundaries which require manual correction of these features. To overcome this issue, we have developed an annotation tool written in python called *AnnoPlast.py.* It uses *Blast+, Biopython* and *pandas* tools for annotation of target sequences from a reference GenBank file. First, all the features from the reference are extracted and then queried against the target sequence using *blastn*. Three rounds of *blastn* are carried out with different percent identities until all the features are mapped. Then the blast output is parsed and annotated to get the target annotations in GenBank format. The *AnnoPlast* annotation tool is also compared with the existing annotation tools such as *GeSeq* Tillich et al., 2017) and *PGA* (Qu et al., 2019). A common practice with newly assembled plastome sequences is to deposit them under the NCBI’s GenBank database. A popular way of doing this is using BankIt which requires annotations in feature table format (suffix.*tbl*). In the final rule, a GenBank to tbl file conversion is carried out to get the feature table file. The progress of each rule is recorded and written into separate log files to debug any errors in the execution of the pipeline. This pipeline is also incorporated with the ability to assemble and annotate multiple plastomes by automatically creating all the required files for all sets of raw reads specified by the user. This would increase the efficiency when many plastomes are assembled.

### Benchmarking

The automated pipeline was tested against three soybean genomes and twelve previously published potato genomes (Achakkagari et al., 2020; Kyriakidou et al., 2020). All soybean and potato plastomes were also manually assembled using the same programs used in the original study (Achakkagari et al., 2020). The protocol for manual assembly includes the command line execution of all the same programs. The timing for all manual or automated assemblies started before the first modification of any file or directory. The timing for all manual or automated assemblies stopped when the jobs finished. All outputs from three repetitions were checked for consistency before declaring the sets of assemblies as successful. All the steps were run on a machine with 180G memory and 16 threads. The parameters for the manual runs were kept same as the original study. The *Trimmomatic* parameters were set to *ILLUMINACLIP : TruSeq3-PE-2.fa:2:30:10 LEADING:3 TRAILING:3 SLIDINGWINDOW:4:15 MINLEN:60*. The parameters used for the *NOVOPlasty* are as follows: assembly type - *chloro*, genome range - *120000 -200000* bp, k-mer size - *29*, max memory - *40G*, read length – *151 bp*, insert size – *300 bp*, and *PE* representing the type of reads as paired-end. The annotation performed by PGA were run with default parameters. The following versions of software were used in all the runs, *Trimmomatic v0.39*, *NOVOPlasty v4.3.1*, *SAMtools v1.13* and *BLAST+ v2.12.0, fastp v0.23.2*. The pipeline was also tested with more complex gymnosperms and angiosperms such as *Cryptocoryne elliptica* (Talkah et al., 2022), *Cyperus rotundus* (Wu et al., 2021b), and *Picea mariana* (Lo et al., 2020).

## Results

Plastid genomes are an essential part of plant cells and play a fundamental role in photosynthesis. Plastomes are highly conserved, and characterization of their sequence help understand the evolutionary relationships among organisms. As more and more sequencing projects are on-going, our pipeline will help to speed up the analysis of plastome sequences. The *Plastaumatic* pipeline has integrated several publicly available and new tools to provide a complete analysis of plastome sequences. The raw reads are processed using *fastp* to solve the issue with finding and providing the adaptor sequences for trimming, since it automatically detects the adaptor sequences. Also, *fastp* has a functionality of subsampling raw reads to a specified amount, which greatly reduces the amount of data to be processed and improves speed. For the assembly, the most popular assembler, *NOVOPlasty*, is used. A new script was developed to solve common standardization issues with assembled plastome sequences. The scrip provides a standardized way of representing the plastome sequences, which is necessary for various downstream analyses. A new annotation tool to accurately annotate all the gene features in the target assembly was also developed. This is to overcome the issues with existing annotation tools improperly annotating gene boundaries or missing some features. And finally, the pipeline integrates a publicly available tool to get an annotation feature table file that is needed for submissions to NCBI’s GenBank. In comparison to other similar pipelines for plastome analysis, *Plastaumatic*, provides more features and features that are essential in plastome analysis and characterization (**Table 1**).

**Table 1:** Comparison of major features of different software for plastome analysis.

Feature	NOVOWrap	GetOrganelle	Fast-Plast	Plastaumatic
Trimming	X	X	✔	✔
*de novo* assembly	✔	✔	✔	✔
Standardization	✔	X	✔	✔
Annotation	X	X	X	✔
Feature table files	X	X	X	✔
Coverage plot	X	✔	✔	X

The *Plastaumatic* pipeline can be executed either as a snakemake pipeline or as a shell script. Publicly available and novel sequences were used to test the *Plastaumatic* pipeline to determine its accuracy and efficiency. All twelve potato plastomes assembled by Plastaumatic were consistent with the manually assembled plastomes and their published plastome assemblies (Achakkagari et al., 2020). The three soybean plastomes assembled by the *Plastaumatic* were also consistent with the three soybean plastomes assembled manually (ON470217-ON470219). The *Cryptocoryne elliptica* assembly is consistent with its published assembly, whereas the *Cyperus rotundus* and *Picea mariana* assemblies have small differences compared with their previously published assemblies (**Table 2**). The *Picea mariana* assembly from the *Plastaumatic* has an additional insertion sequence of 28 bp, which likely resulted from different assembly methods used. Though the *Cyperus rotundus* assembly obtained from this study is longer than the published assembly, it is more consistent with plastomes from other *Cyperus* species. Hence it is highly likely that the original assembly of *Cyperus rotundus* is incomplete.

**Table 2:** List of species used in testing the pipeline.

Species	Taxonomy	SRA	GenBank	Size (original study)	Size (*Plastaumatic*)	Percent Identity
*Solanum stenotomum* subsp*. goniocalyx*	eudicots	SRR10244441	MT120855	155,492	155,492	100
*Solanum stenotomum* subsp*. goniocalyx*	eudicots	SRR10244440	MT120856	155,492	155,492	100
*Solanum xajanhuiri*	eudicots	SRR10244437	MT120857	155,486	155,486	100
*Solanum phureja*	eudicots	SRR10244439	MT120858	155,492	155,492	100
*Solanum stenotomum* subsp*. stenotomum*	eudicots	SRR10244438	MT120859	155,492	155,492	100
*Solanum bukasovii*	eudicots	SRR10244436	MT120860	155,491	155,491	100
*Solanum juzepczukii*	eudicots	SRR10248512	MT120863	155,532	155,532	100
*Solanum chaucha*	eudicots	SRR10248511	MT120864	155,518	155,518	100
*Solanum tuberosum* subsp*. andigena*	eudicots	SRR10248515	MT120861	155,530	155,530	100
*Solanum tuberosum* subsp*. andigena*	eudicots	SRR10248514	MT120862	155,518	155,518	100
*Solanum tuberosum* subsp*. tuberosum*	eudicots	SRR10248513	MT120865	155,564	155,564	100
*Solanum curtilobum*	eudicots	SRR10248510	MT120866	155,492	155,492	100
*Cryptocoryne elliptica*	monocots	SRR14784941	MZ435316.1	159,968	159,968	100
*Picea mariana*	conifers	SRR12885547	MT261462.1	123,961	123,986	99.97
*Cyperus rotundus*	monocots	SRR12799673	MT937176.1	182,986	186,127	100
Glycine max^*^	eudicots	SRR19105742	ON470219	–	152,226	–
Glycine max^*^	eudicots	SRR19103585	ON470217	–	152,226	–
Glycine max^*^	eudicots	SRS6529047	ON470218	–	152,226	–

A table listing all the species used in this study to test the pipeline and their SRA and GenBank accession numbers. The plastome assembly size from the original study and this study are compared. Species marked with ^*^ are novel plastome assemblies generated using the Plastaumatic pipeline.

The annotations obtained from the *Plastaumatic* for each genome are the same as their original annotations. All the gene features were correctly annotated through *Plastaumatic*. The accuracy of annotations was also compared with the other available tools such as GeSeq and PGA. While the GeSeq performed better than PGA, it incorrectly annotated some gene features. The *rps12* gene is a trans-splicing gene and it is often difficult to annotate. GeSeq was unable to properly annotate the *rps12* gene and other genes such as *petB*, *petD*, and *rpl16*. The *PGA* tool also annotated the *rps12* gene incorrectly, along with *ycf3*, *ndhD* genes, and any *trnA* with a short length. Also, PGA does not report intron and exon features in the output GenBank file. In comparison to these tools, the *AnnoPlast* performs better and generates accurate gene features.

### Twelve potato plastomes (for which plastome assemblies are previously published)

The time taken to assemble twelve potato plastomes manually was measured separately for each of the three repetitions to be 373, 281, and 336 minutes, respectively, resulting in an average time of 330 minutes with a peak memory usage of 40G. The time taken to assemble the twelve potato plastomes using the automated pipeline were 36, 36, and 36 minutes, resulting in an average assembly time of 36 minutes with a peak memory usage of 11G. The automated pipeline finished ~10x faster compared to the manual assembly with only ¼^th^ of memory. Thus, *Plastaumatic* means a significant decrease in the time taken to finish the plastome assembly and annotations compared to the manual assembly.

### Three soybean plastomes (not previously published)

The time taken to assemble three soybean plastomes manually, measured separately for each of the three repetitions, were 202, 215, and 182 minutes, respectively, resulting in an average time of 200 minutes with a peak memory usage of 40G. The time taken to assemble three soybean plastomes using the automated pipeline with three repetitions, were 28, 26, and 27 minutes resulting in an average assembly time of 27 minutes with a peak memory usage of 11G. Similar to the results with the potato genomes, the *Plastaumatic* finished ~7x faster compared to the manual assembly with about ¼^th^ of memory. These soybean plastomes assemblies were submitted to the NCBI GenBank under the accession numbers ON470217-ON470219.

## Discussion

### Plastaumatic pipeline performance

For both the twelve potato genomes and the three soybean genomes we could confidently conclude that adopting the automated pipeline resulted in substantial decrease in time and memory needed for complete assembly and annotation, thus a huge increase in efficiency. The time taken for manual assembly were less consistent compared to the automated assembly using the pipeline. One of the factors contributing to such inconsistency was the amount manual inspection needed after steps such as annotation. In the twelve assembled and annotated potato plastomes, the *ycf3* gene feature was reported to contain internal stop codons. In all assembled and annotated soybean plastomes, the *ndhB* gene feature was reported to contain internal stop codons. Such results are due to error made by *PGA* during annotations. The detection of internal stop codons in assemblies usually calls for corrections made to the corresponding coordinates manually though *blastn* searches. As previously introduced, this adopted workflow of plastome assembly consisted of six different programs to complete. Working on high-performance computing systems and executing each of the six programs would require various specifications, including but not limited to input and output full paths, containing directories, accessory references, or configurations. In the meantime, while the required user input information was overall not complicated, the forms of the paths or files requested by the six programs were not unified to optimize the compatibility of each tool to the others. Therefore, inputting information for each program would require more effort than copying and pasting the same texts from the previous step. When the number of genomes to be assembled is increased, the time needed for repeated inspections on whether the input information was correctly entered was substantially elevated. Such impact could be seen from the rather significant difference between the time taken for manual assembly of the three soybean plastomes and that of the twelve potato plastomes.

In all manual assemblies of plastomes, the user must wait for an upstream job, e.g., Trimmomatic, to finish before the downstream job, e.g., NOVOPlasty, could be submitted, since manual execution of each program requires indication of paths of the input files, which could not be known before the outputs were produced by the upstream program. Therefore, by the nature of doing manual plastome assemblies, some periods of time in between the execution of the programs would be wasted if the user did not get the notification message of an upstream program finishing, thus creating lag between connections and lengthening the overall time needed for manual plastome assemblies.

### Limitations

Limitations to the pipeline are currently issues inherent from component software and are the same as with manual processing. For example, when the *de novo* assembly by *NOVOPlasty* is not finished properly, it yields multiple suggested assembled plastomes where the program was unable to decide which one is the most appropriate assembly. Since the number of outcomes from *NOVOPlasty* hardly exceeds two, the situation where more than two outcomes were produced by *NOVOPlasty* results the pipeline to exit. This can be controlled by providing a reference *fasta* sequence for *NOVOPlasty*, that is providing the path to a reference sequence in *fasta* format in the *NOVOPlasty* configuration file.

### Comparison with existing tools

Overall, there has not been many attempts to automate the workflow of plastome assembly and annotation. One program with similar motivations was called *NOVOWrap* (Wu et al., 2021a). *NOVOWrap* was designed to automatically assemble, validate, and standardize plastomes with minimum inputs and user intervention. While such objectives sound similar to the *Plastaumatic* pipeline, they differ on many aspects. Firstly, with respect to the workflow of plastome assembly and annotation in the current study, the usage of *NOVOWrap* would only achieve partial automation. Users would still have to pre-process reads to remove adapter sequences to use as an input for *NOVOWrap*. Secondly, since *NOVOWrap* only provides functions of *de novo* assembly, validation and standardization, the final outputs of *NOVOWrap* would not be annotated, unlike the output of *Plastaumatic*, and would therefore require further processes before being NCBI publication ready. Finally, while both the *Plastaumatic* pipeline and *NOVOWrap* proposed automatic assembly of plastomes, our intentions were different enough to cause the two programs to be optimized in completely different directions. The ultimate aim for our automated pipeline was to eliminate manual input thus achieving higher efficiency in batch assembly and annotation of large numbers of plastomes altogether. Naturally, all parameters specified in our automated pipeline aimed to require the least amount of time when processing and is optimized to run on multiple genomes. In conclusion, while parts of our automated pipeline and *NOVOWrap* share similarities, they are optimized to perform very different types of assembly tasks, and *Plastaumatic* is well suited for complete plastome batch assembly and annotation with an NCBI-ready final output.

## Data availability statement

Publicly available datasets were analyzed in this study. The sequencing data presented in the study are deposited in the NCBI repository (https://www.ncbi.nlm.nih.gov/bioproject/), the BioProject accession numbers are PRJNA556263, PRJNA835403, PRJNA627639, and PRJNA835489. The plastome assemblies presented in the study are deposited in the GenBank repository (https://www.ncbi.nlm.nih.gov/nuccore/), accession numbers are MT120855.1 - MT120866.1, and ON470217-ON470219.

## Author contributions

WC, SA, and MS conceptualized the project, WC and SA developed the pipeline, drafted and edited the manuscript, MVS supervised the project and edited the manuscript. All authors contributed to the article and approved the submitted version.

## Funding

Funding for the project was provided through a Compute/Calcul Canada Resource Allocations for Research Portals and Platforms (The Potato Genome Diversity Portal) award and a Génome Québec award (GQ-AAC-2019-2) to MVS. Sequencing of the soybean plastomes was funded by a Discovery Grant from the Natural Sciences and Engineering Research Council of Canada (NSERC) to MVS.

## Acknowledgments

The authors wish to thank Ilayda Bozan for technical help.

## Conflict of interest

The authors declare that the research was conducted in the absence of any commercial or financial relationships that could be construed as a potential conflict of interest.

## Publisher’s note

All claims expressed in this article are solely those of the authors and do not necessarily represent those of their affiliated organizations, or those of the publisher, the editors and the reviewers. Any product that may be evaluated in this article, or claim that may be made by its manufacturer, is not guaranteed or endorsed by the publisher.

## References

[B1] Achakkagari S. R. Kyriakidou M. Tai H. H. Anglin N. L. Ellis D. Strömvik M.V. (2020). Complete plastome assemblies from a panel of 13 diverse potato taxa. PloS One 15 (10), e0240124. doi: 10.1371/journal.pone.0240124 33031462 PMC7544113

[B2] Achakkagari S. R. Tai H. H. Davidson C. Jong H. Strömvik M. V. (2021). The complete plastome sequences of nine diploid potato clones. Mitochondrial DNA B Resour. 6 (3), 811–813. doi: 10.1080/23802359.2021.1883486 33763586 PMC7954508

[B3] Bolger A. M. Lohse M. Usadel B. (2014). Trimmomatic: a flexible trimmer for Illumina sequence data. Bioinformatics 30 (15), 2114–2120. doi: 10.1093/bioinformatics/btu170 24695404 PMC4103590

[B4] Camacho C. Coulouris G. Avagyan V. Ma N. Papadoupoulos J. Bealer K. . (2009). BLAST+: Architecture and applications. BMC Bioinf. 10, 421. doi: 10.1186/1471-2105-10-421 PMC2803857 20003500

[B5] Camargo Tavares J. C. Achakkagari S. Archambault A. Strömvik M. V. (2022). The plastome of the arctic oxytropis arctobia (Fabaceae) has large differences compared with that of o. splendens and those of related species. Genome 65 (5), 301–313. doi: 10.1139/gen-2021-0059 35245153

[B6] Chen S. Zhou Y. Chen Y. Gu J. (2018). Fastp: An ultra-fast all-in-one FASTQ preprocessor. Bioinformatics 34 (17), i884–i890. doi: 10.1093/bioinformatics/bty560 30423086 PMC6129281

[B7] Chung H.-J. Jung J. D. Park H.-W. Kim J.-H. Cha H. W. Min S. R. . (2006). The complete chloroplast genome sequences of solanum tuberosum and comparative analysis with solanaceae species identified the presence of a 241-bp deletion in cultivated potato chloroplast DNA sequence. Plant Cell Rep. 25 (12), 1369–1379. doi: 10.1007/s00299-006-0196-4 16835751

[B8] Danecek P. Bonfield J. K. Liddle J. Marshall J. Ohan V. Pollard M. O. . (2021). Twelve years of SAMtools and BCFtools (Gigascience), Vol. 10, giab008. doi: 10.1093/gigascience/giab008 PMC7931819 33590861

[B9] Dierckxsens N. Mardulyn P. Smits G. (2017). NOVOPlasty: *de novo* assembly of organelle genomes from whole genome data. Nucleic Acids Res. 45 (4), e18. doi: 10.1093/nar/gkw955 28204566 PMC5389512

[B10] Hoopes G. Meng X. Hamilton J. Achakkagari S. R. Alves Freitas Guesdes F. Bolger M. E. . (2022). Phased, chromosome-scale genome assemblies of tetraploid potato reveals a complex genome, transcriptome, and proteome landscape that underpin phenotypic diversity. Mol. Plant 15 (3), 520–536. doi: 10.1016/j.molp.2022.01.003 35026436

[B11] Jin J. J. Yu W. B. Yang J. B. Song Y. DePamphilis C. W. Yi T. S. . (2020). GetOrganelle: A fast and versatile toolkit for accurate *de novo* assembly of organelle genomes. Genome Biol. 21, 241. doi: 10.1186/s13059-020-02154-5 32912315 PMC7488116

[B12] Kyriakidou M. Achakkagari S. R. Galvez J. H. Zhu X. Tang K. Tai H. . (2020). Structural genome analysis in potato taxa. Theor. Appl. Genet. (TAG) 133, 951–966. doi: 10.1007/s00122-019-03519-6 31893289 PMC7021743

[B13] Lo T. Coombe L. Lin D. Warren R. L. Kirk H. Pandoh P. . (2020). Complete chloroplast genome sequence of a black spruce (Picea mariana) from Eastern Canada. Microbiol. Resour. Announcements 9 (39), e00877–e00820. doi: 10.1128/MRA.00877-20 PMC7516155 32972944

[B14] McCauley D. E. Sundby A. K. Bailey M. F. Welch M. E. (2007). Inheritance of chloroplast DNA is not strictly maternal in silene vulgaris (Caryophyllaceae): evidence from experimental crosses and natural populations. Am. J. Bot. 94 (8), 1333–1337. doi: 10.3732/ajb.94.8.1333 21636500

[B15] McKain M. R. Wilson M. (2017) Fast-plast: rapid de novo assembly and finishing for whole chloroplast genomes. Available at: https://github.com/mrmckain/Fast-Plast.

[B16] Mölder F. Jablonski K. P. Letcher B. Hall M. B. Tomkins-Tinch C. H. Sochat V. . (2021). Sustainable data analysis with snakemake [version 1; peer review: 1 approved, 1 approved with reservations]. F1000Research 10, 33. doi: 10.12688/f1000research.29032.1 34035898 PMC8114187

[B17] Qu X. J. Moore M. J. Li D. Z. Yi T. S. (2019). PGA: a software package for rapid, accurate, and flexible batch annotation of plastomes. Plant Methods 15, 50. doi: 10.1186/s13007-019-0435-7 31139240 PMC6528300

[B18] Talkah N. S. M. Wongso S. Othman A. S. (2022). Complete chloroplast genome data for cryptocoryne elliptica (Araceae) from peninsular Malaysia. Data Brief. 42, 108075. doi: 10.1016/j.dib.2022.108075 35392620 PMC8980536

[B19] Tillich M. Lehwark P. Pellizzer T. Ulbricht-Jones E. S. Fischer A. Bock R. . (2017). GeSeq – versatile and accurate annotation of organelle genomes. Nucleic Acids Res. 45, W6–W11. doi: 10.1093/nar/gkx391 28486635 PMC5570176

[B20] Wu P. Xu C. Chen H. Yang J. Zhang X. Zhou S. (2021a). NOVOWrap: An automated solution for plastid genome assembly and structure standardization. Mol. Ecol. Resour. 21 (6), 2177–2186. doi: 10.1111/1755-0998.13410 33934526

[B21] Wu R. Yu C. Wu Y. (2021b). Characterization of the complete plastome of cyperus rotundus l. (Cyperaceae). Mitochondrial DNA Part B 6 (1), 58–59. doi: 10.1080/23802359.2020.1845999 33521266 PMC7819115

